# Intercropping maize (*Zea mays* L.) and field beans (*Vicia faba* L.) for forage, increases protein production

**DOI:** 10.1038/s41598-024-67091-w

**Published:** 2024-07-16

**Authors:** Józef Sowiński

**Affiliations:** Institute of Agroecology and Crop Production, Wroclaw University of Environmental and Life Sciences, 50-375, Wrocław, Poland

**Keywords:** Maize–faba bean agroecology system, Growth dynamics, Yield, Chemical composition, Protein–energy balance, Biochemistry, Plant sciences

## Abstract

In 2005–2007, a field study was conducted into intercropping of maize with faba bean at Pawlowice research station, Wroclaw University of Environmental and Life Sciences. The main aim of the multi-year field research was an investigation into the reactions of differing maize hybrid earliness to intercropping cultivation with faba bean. The field research evaluated the effect of three maize hybrids—Wilga (early—E), Blask (medium—M) and Iman (late—L)—and the sowing rate of faba bean—18 (Fb1), 27 (Fb2) and 36 (Fb3) seeds per 1 m^2^—on growth dynamics and yield structure, and biomass, protein, and energy yield. Cultivation of faba bean in maize inter-rows led to significant competition with maize and affected yields, causing a decrease in maize dry matter yield from 14.1 (Fb1) to 20.6% (FB3) compared with maize sown alone. In terms of total biomass yield from maize and faba beans, no significant differences were found, but a slight increase in yield of 1.1–4.2% (repective to Fb1 and Fb3) was noted compared to maize sown alone. The early maize hybrid had a significantly lower yield but was most suitable for intercropping with faba bean. The dry biomass yield of early hybrids increased in intercropping by 25% compared to pure maize cultivation. Total protein yield from both intercropping components was higher than in the pure sowing of maize: from 24 (Fb1) to 39% (Fb3). The increase in protein production resulted in an improvement in the energy–protein ratio. The number of UFL per kg of total protein decreased from 13.2 in pure maize cultivation (M-P) to 9.3 (Fb3). A more balanced forage biomass was produced from intercropping maize with faba bean, especially when an early maize hybrid was sown with faba beans.

## Introduction

Global transformation processes especially industrialization exert an effect on climate changes. Temperature increases and the negative impact of this process are the main reasons for the introduction of supporting programs for environmentally friendly food production systems^1^. The implementation of Green Deal strategies in line with agroecology principles and regenerative farming will involve an increase in the importance of leguminous crops^2–4^. This is due to the beneficial effects of these species in reducing Greenhouse Gas (GHG) emissions as well as the consumption of non-renewable energy resources^5^. The cultivation range of legume species can also be expanded through the dissemination of intercropping^1,6^.

Studies on the intercropping of leguminous crops with cereals for whole-crop biomass or grain have previously been reported^7–13^. Some authors indicate that harvesting forage from an intercropping system provides a better balance of energy and protein biomass. However, limited information is available for intercropping maize with legumes in moderate climates. In most instances, the cultivation of maize in pure sowing has been evaluated^14,15^. Several studies have demonstrated its adverse effects on soil properties^15^. Intercropping with legume species, increasing the soil shading between the rows and by a deep, tap root system can reduce the negative impact of crops on the environment^15–22^. Even though maize is a cereal species, it has different requirements than other cereals regarding, for example, specific plant spaces for development growth and morphology. Therefore, research is necessary to determine the impact of selected species on the biomass, protein, and energy yield.

Forage quality, along with yield and economic efficiency are the main criteria under consideration in modern forage production systems. Forages with high energy value and high starch content are essential for milk and livestock production, especially in high-output systems. Maize is one of the most significant crops in the world, predominantly serving the needs of dairy and livestock producers^23^. Maize biomass at milk-dough maturity stage is not balanced regarding energy and protein parameters. The protein content depends on the development stage and generally is too low, ranging from 6% (at grain maturity) to 12% in Dry Matter (DM) (at vegetative growth). In addition, maize protein is characterized by low digestibility: approximately 50%^24^. Therefore, in the feed of high-yielding ruminants, maize silage must be supplemented with legume protein concentrates or ensilaged with legume species or other protein-rich forages.

Maize is cultivated in wide inter-rows. In the initial growing period, it develops slowly and is not competitive with other plants. The free space between the rows can be successfully used to grow legumes as an intercrop species. In intercropping, it is crucial that the implemented system does not compete with the maize. Additionally, there must be an appropriate selection of herbicides to enable inter-row cultivation of maize with the other species.

Climate change—an effect of the exploitation of fossil fuels—also modifies crop cultivation technology. Higher temperatures and longer growing seasons contribute to search new agronomy solutions. Early maize hybrids can be sown as the second crop in the same production season (e.g. after winter barley, early potatoes or peas grown for green seeds as a vegetable). Cultivation is possible due to the extension of the vegetation period and the late-autumn occurrence of frost. On the other hand, reduction of the consumption of fossil fuels can be achieved by using legume species. Intercropping of maize with faba beans is possible due to climate change, but at the same time this system can reduce the negative impact of agricultural production on climate change.

The main aim of this research was an investigation of the reaction of maize hybrids of differing earliness to an intercropping system with faba bean for production of a more energy–protein balanced forage. The competitiveness of faba bean was assessed according to different sowing rates. The energy–protein balance was estimated with the UFL–protein ratio (UFL—Unité Fourragère Lait).

The study adopted the following working hypotheses: (1) maize hybrids of varying earliness will respond differently to intercropping with faba bean sown in different densities; and (2) competition between the intercropped species will result in biomass yields, while improving protein yield and the energy–protein balance of the harvested biomass.

## Materials and methods

### Field experiment

#### Experiment location and design

A 3-year field study was carried out in 2005–2007 at the Pawłowice research station, Wroclaw University of Environmental and Life Sciences. The experiment was established on a Stagnic Luvisol soil from sandy loam on loam. This soil was characterized by a slightly acid reaction (pH in the range of 5.6–5.7), an average phosphorus content (70.7–87.3 mg kg^−1^ of soil), an average potassium content (121.6–127.3 mg kg^−1^ of soil), and a very high magnesium content (124.5–151.6 mg kg^−1^ soil). The 3-year experiment was organized using the randomized sub-block method with two variable factors: hybrid earliness [Wilga FAO 190—early hybrid (E), Blask FAO 250—medium hybrid (M), Iman FAO 290—late hybrid (L)], and sowing method [maize in pure sowing (M-P) and intercropping of maize with faba bean between maize rows]. The sowing rates of the faba bean varied: 18 seeds (Fb1), 27 seeds (Fb2), and 36 seeds per m^2^ (Fb3). The maize sowing rate (irrespective of sowing methods and hybrids) was nine grains per m^2^ sown in 70 cm inter-rows. The experiment was conducted in four replications. Maize and faba bean were sown on 27, 29, and 25 April in 2005, 2006, and 2007, respectively. In the research, cultivated crops were treated as they would be in farming practice. According to national regulations, the use of maize and faba beans for experimental purposes does not require any specific permit. This study complies with relevant institutional, national, and international guidelines and legislation.

#### Agrotechnical management

**Figure 1:**
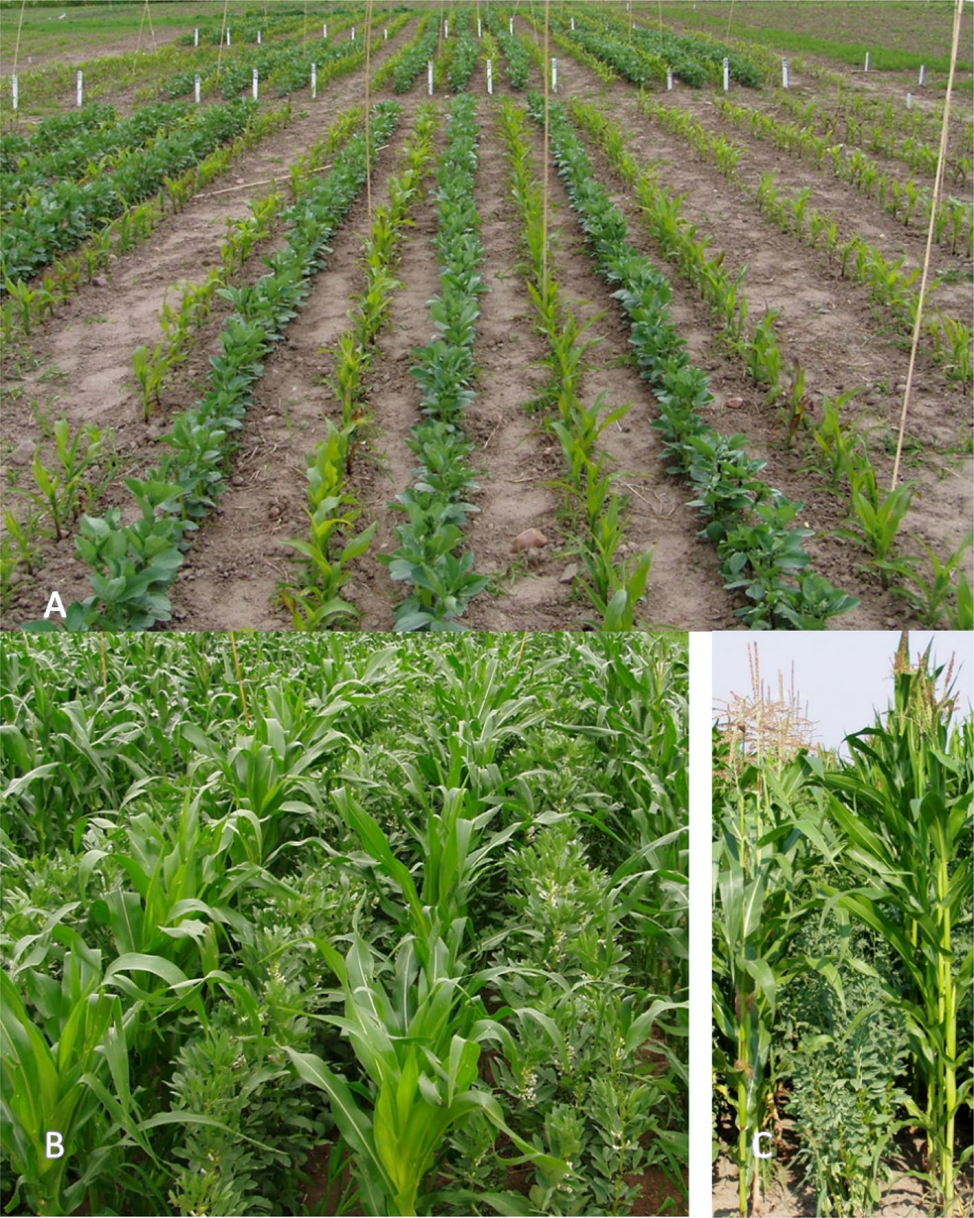
Maize—faba bean intercropping. (**A**) Before measurement date I, (**B**) at measurement date II, (**C**) at measurement date V.

The experiment was established following winter wheat. Ploughing was performed in the first half of November in the autumn of the year preceding the start of the experiment. In spring, after levelling the field, fertilizers were applied at the following rates: N—100 kg ha^−1^ in the form of urea (46%), P_2_O_5_—90 kg ha^−1^ as triple superphosphate (46%), and K_2_O—120 kg ha^−1^ as potassium salt (60%). The dose of nitrogen fertilizer was 30% lower than the recommended range in the study region. The doses of phosphorus and potassium were determined according to the average concentration of these macronutrients in the soil. Upon application, the fertilizers were mixed with the soil using an active aggregate (rotary harrow + tine roller). Sowing of both species was conducted by hand in the third decade of April, on the same date for both species. Faba bean seeds of the *Nadwiślański* (open-pollinated varieties) were sown in the middle of maize inter-rows, at a distance of 35 cm from the maize rows. The faba bean seeds per unit area varied according to the second experimental factor. Immediately after sowing, linuron was applied at a rate of 0.9 l active ingredients ha^−1^. No additional herbicides were sprayed during the growing season, and weeds were removed by hand. The plot area was 7 m^2^ (Fig. 1).

#### Plant morphology measurement

Plant height measurements were taken during the growing season at growing stages relevant for maize (BBCH): 13—I measurement, 17—II measurement, 33—III measurement, 51—IV measurement, and 69—V measurement^25^. Biomass composition analysis (percentage of maize and faba beans in whole forage biomass, percentage of cobs and stover of maize, and percentage of steam and pods of faba beans) of the intercropping components was performed before harvest. Harvesting was performed manually at milk-dough stage, and the fresh crop yield was determined separately for each species. Based on the yield and dry matter content, the dry matter yield per ha was calculated.

#### Chemical analysis

Chemical analysis and forage quality parameter calculation

In the harvested plant material, analyses of the chemical composition of the forage were performed for each component:

- dry matter, obtained by the oven-drying method at 105 °C
- total protein, by the Kjeldahl method after mineralization with sulfuric acid and perhydrol
- crude fat, by the Soxhlet method after extraction with ethyl ether
- crude fiber, by the Hennenberg–Stohmann method
- crude ash, incineration in an oven at 600 °C.

Based on the percentage of each species and the chemical composition, the weighted average content of the components was calculated (Eq. 1):

1 $${\overline{x} }_{wi}= \frac{{{(\text{M}}_{\text{p}} \cdot \text{ M}}_{\text{ic}})+({\text{Fb}}_{\text{p}} \cdot {\text{Fb}}_{\text{ic}})}{{\text{M}}_{\text{p}}+{\text{Fb}}_{\text{p}}}$$

${\overline{x} }_{wi}$—weighted mean of compounds *i*, Mp**—**maize proportion, Mic**—**maize compound *i* concentration, Fbp**—**faba bean proportion, Fbic**—**faba bean compound *i* concentration.

On the basis of the yield volume and the weighted average of protein content, the protein yield was calculated.

#### Forage quality calculation

The energy value of forage is presented in units of milk production (*Unité Fourragère Lait*—UFL)^26^. In determining the energy value of forage in UFL units, 1 kg of barley grain was used as a reference forage. The UFL is calculated based on the net energy lactation (NEL) in kcal kg^−1^ dry matter. The calculation of NEL is based on the chemical composition of the forage (total protein, crude fat, crude fiber, N-free extract) and the digestibility of the mentioned components in the forage. The resulting NEL is divided by the factor of 1700 to obtain the feed energy value unit expressed in UFL per kg of dry matter^26^.

For the biomass of whole maize plant biomass harvested at milk-dough maturity, the digestibility coefficients were 42, 74, 70, and 79% for total protein, crude fat, crude fiber and N-free extract, respectively. The digestibility coefficients for whole faba bean at full maturity were 65, 64, 53, and 78%, respectively. The energy yield of the forage at UFL was calculated from the dry matter yield and energy value of 1 kg DM.

Based on the energy yield and protein yield, the forage balance index was calculated as the number of UFL per kg of total protein.

### Weather conditions

**Figure 2:**
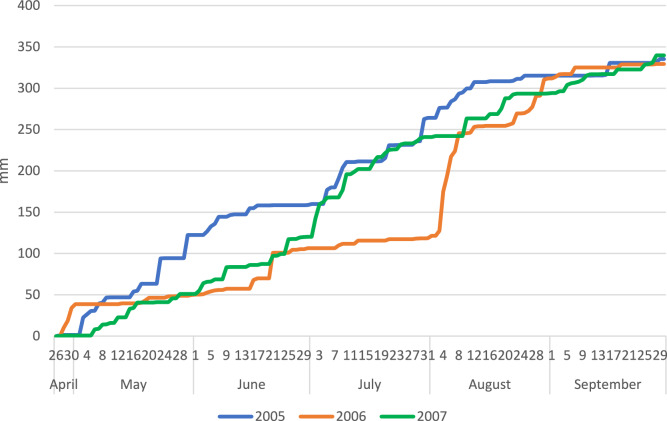
Cumulative rainfall (mm) during vegetation periods of maize and faba bean.

The distribution of rainfall during the study years differed from the optimum for maize. In 2005 and 2007, in the period from the beginning of May to the end of the 1st decade of July, the total rainfall was more than 100% higher than the optimum for this period, while in 2006 it was lower, amounting to 77% of the optimum level (Fig. 2). In the latter of the growing periods, the rainfall was below the optimum for maize in all years of the study. In 2006 and 2007, the rainfall during the generative development period of maize was below the optimum values for this region.

The rainfall distribution during the period of the study was not favorable for the development of faba bean. In May 2005, optimum rainfall levels were exceeded by more than 50%, while in 2006 and 2007 they accounted for only 20% of the optimum level. The rainfall deficit was particularly noticeable in June and July 2006, constituting only 36.3% of the optimum for that period. In 2007, a large volume of rainfall occurred in July, exceeding the optimum value for this species by 50%.

For thermal conditions, the accumulated growing degree days (AGDD) were calculated (Eq. 2):

2 $$AGDD= \sum_{i=0}^{n}(T_{average}-T_{base})$$

AGDD—accumulated growing degree days, T_average_—average daily temperature, T_base_—for maize 10°C.

**Figure 3:**
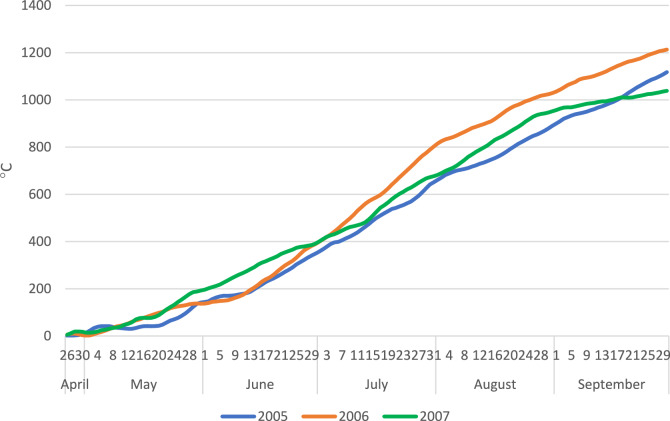
Accumulated growing degree days (°C) during vegetation periods of maize and faba bean.

Thermal conditions in periods of maize and faba bean growth were at or above the optimum temperature for these species (Fig. 3). During the growing season of intercropped species, temperature exerted no stress effect on the development.

### Statistical analysis

Measurements obtained from maize and faba beans during the study were statistically processed using ANOVA analysis of variance, with verification of the significance of differences between group averages using Tukey's multiple range test with Statistica 13.3 software. Both one-way analyses and two-way analyses across years were employed to assess the effects of selected maize hybrids and sowing method on specific dependent variables. For maize, faba bean, and total protein and energy yield three-way ANOVA was employed, with years as fixed factors.

## Results

### Effect of intercropping system on maize and faba bean growth

**Figure 4:**
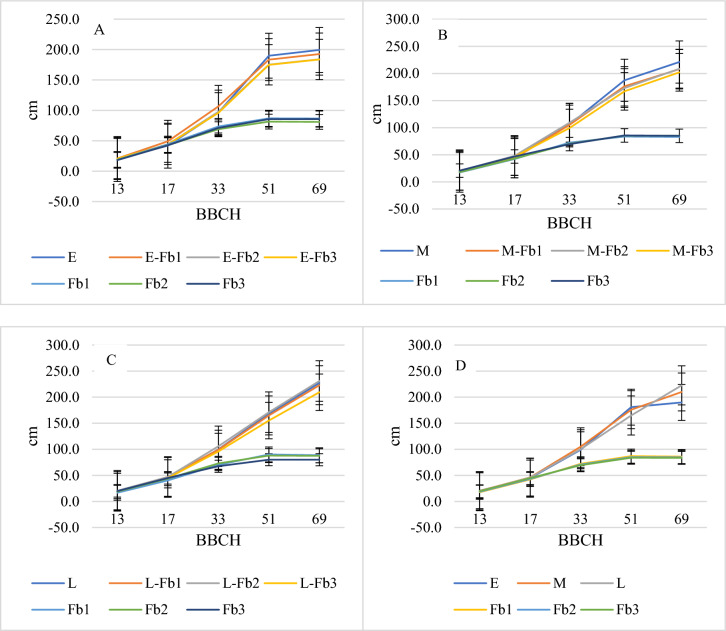
Maize and faba bean plant height. Average data from 2005 to 2007. (**A**) Early hybrid with faba beans; (**B**) medium hybrid with faba beans; (**C**) late hybrid with faba beans; (**D**) average for hybrid and faba bean sowing rates; E—early hybrid; M – medium hybrid; L—late hybrid; Fb1—faba beans, sowing rate 18 seeds m^−2^; Fb2—faba beans, sowing rate 27 seeds m^−2^; Fb3—faba beans, sowing rate 36 seeds m^−2^.

In the initial period (on the first and second measurement dates), the growth dynamics of maize and faba bean were similar (Figs. 1, 4). No competition between species was recorded at this period. From the third measurement date, the growth of maize increased, while faba bean growth stabilized at the height of 80–90 cm. Maize in pure sowing was highest at the end of the measurement period (Fig. 4A–C). The competition effect of the sowing rate of faba bean on maize growth was similar, irrespective of the hybrid. Increasing the faba bean sowing rate from 18 to 36 seeds per 1 m^2^ had a decreasing effect on maize height. However, increasing the sowing rate of faba bean from 18 to 36 seeds per 1 m^2^ had no effect on the legume plant height. The hybrid type (early and medium) showed similar growth dynamics up to maize 51 BBCH growing stages. In the same period, the growth of the late hybrid was the lowest. Late hybrids started intensive growth compared to other hybrids later in more stressful water-deficit conditions. In the last measurement (at stage BBCH 69), high differences were recorded between hybrids. The Wilga hybrid had an average height of 189 cm, Blask 210 cm, and Iman 223 cm. The average plant height of all maize hybrids, as an effect of faba bean sowing rates, showed no differences (Fig. 4D).

### Effect of intercropping system on maize and faba bean biomass yield

**Figure 5:**
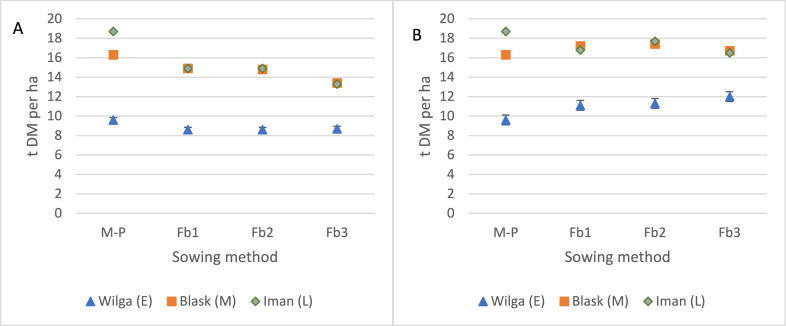
Dry matter yield of maize (**A**) and intercropping maize with faba bean (**B**) (t per ha). Interaction of hybrids and sowing method. Mean for 2005–2007. M-P—maize pure sowin;, E—early hybrid; M—medium hybrid; L—late hybrid; Fb1—faba beans, sowing rate 18 seeds m^−2^; Fb2—faba beans, sowing rate 27 seeds m^−2^; Fb3—faba beans sowing rate 36 seeds m^−2^.

In all hybrids, the highest dry matter yield was found when maize was cultivated without the faba beans (Table 1). The decrease in the yield of the hybrids varied in relation to the increasing proportion of faba bean (*p* < 0.001). In the Wilga hybrid, dry matter yield in intercropping was from 9.5 (Fb3) to 10.1% (Fb1, Fb2) lower than pure sowing of an early hybrid (Fig. 5A). The Blask hybrid, when intercropped with faba bean, had a 9.1 (Fb1) to 17.9% (Fb3) lower yield than in pure sowing. The late hybrid Iman reacted most strongly to faba bean competition, and the DM yield was 20.6 (Fb1) to 28.9% (Fb3) lower than that from maize without a legume crop. Maize hybrid selection for intercropping system with faba bean was crucial for biomass yield. Fast growing early and medium maize hybrids adapted better to the legume competition than did later ones.

**Table 1:** Variance analysis of maize dry matter yield.

Factor	Sum of squares	df	Mean square	F	Significance
Sowing methods	18,172.8	3	6057.6	6.3	< 0.001***
Hybrid	126,625.5	2	63,312.7	65.4	< 0.001***
Year	37,495.1	2	18,747.6	12.3	0.001***
Sowing methods x hybrid	6759.1	6	1126.5	1.2	0.329
Year × sowing methods	22,560.3	6	3760.1	2.5	0.027*
Error: year × sowing methods	201,037.8	132	1523	–	–
Year × hybrid	17,498.1	4	4374.5	6.0	0.001***
Error: year × hybrids	97,647	135	723	–	–
Error	35,519.2	108	328.9	–	–

Significant differences were found between the hybrids (*p* < 0.001) (Table 2) for total yield of maize and faba beans. For the hybrids Wilga and Blask in an intercropping system, regardless of the sowing rate for faba bean, provided a total DM yield of both components higher than that in pure sowing (Fig. 5B). No similar tendency was shown with the Iman hybrid. By intercropping the early hybrid with faba bean, a yield increase occurred with increasing sowing rate for the faba bean plant. The highest increase (25.1%) was obtained with Fb3. With the medium hybrid, the highest yield increase was noted (6%) when the sowing rate was increased to 27 faba bean seeds per 1 m^2^ (Fb2). For the late hybrid, yield decreased from 5.3% (FB2) to 11.8% (FB3) compared to pure sowing of the Iman hybrid.

**Table 2:** Variance analysis of dry matter yield of maize and faba bean intercropping.

Factor	Sum of squares	df	Mean square	F	Significance
Sowing methods	745.4	3	248.5	0.3	0.828
Hybrid	122,318.0	2	61,159.0	73.0	< 0.001***
Year	23,501.8	2	11,750.9	7.9	0.001***
Sowing methods × hybrid	7440.2	6	1240.0	1.5	0.190
Year × sowing methods	20,857.7	6	3476.3	2.3	0.035*
Error: year × sowing methods	196,093	132	1486		
Year × hybrid	14,233.5	4	3558.4	5.9	0.001***
Error: year × hybrids	81,128	135	601		
Error	37,236.8	108	344.8		

**Figure 6:**
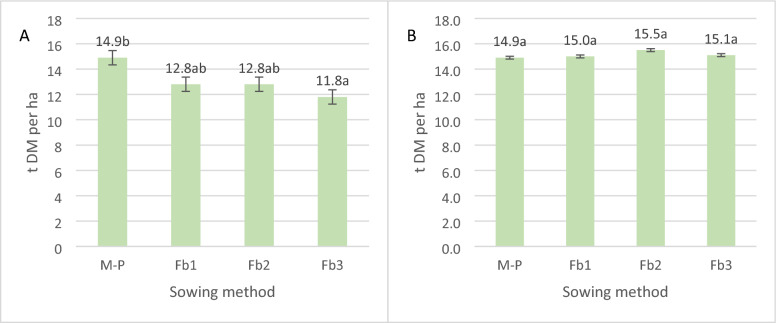
Dry matter yield of maize (**A**) and intercropping maize with faba bean (**B**) (t per ha). Average for sowing method. Mean for 2005–2007.

There were significant differences in biomass yield of maize (Fig. 6A) but no significant differences in the total biomass yield of maize and faba bean dependent on the sowing method (Fig. 6B). Faba bean biomass yield had no effect on total biomass yield nor did it lead to differences between hybrids. The early maize hybrid with faba bean produced a significantly lower yield than did the others.

It is important to highlight the reduction in the difference of total maize yield with faba bean compared to the differences between the maize hybrids in terms of yield. The biomass yield from intercropping compared to the difference between maize hybrids only, for Iman (highest yielding) and Wilga (lowest), decreased by 15.5 percentage points.

### Effect of intercropping system on maize and faba bean biomass structure

**Figure 7:**
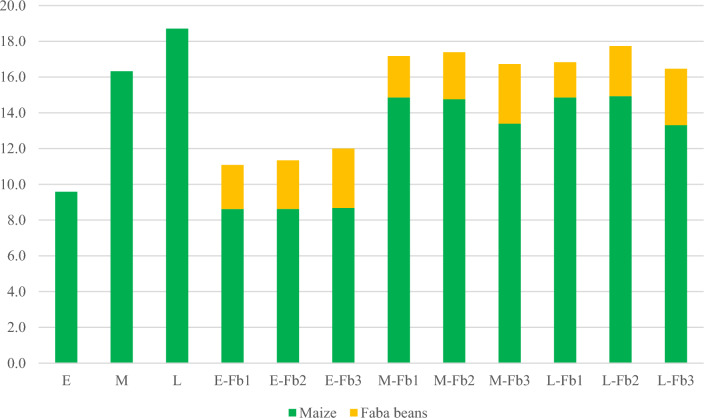
Structure of harvested biomass (in t of D.M. per ha). Percentage of maize and faba bean biomass.

The choice of hybrid and the sowing rate of the faba bean had an effect on the percentage of biomass components at harvest stage (Fig. 7). The Wilga hybrid was the least competitive, and the share of faba bean in total biomass yield ranged from 22.3 to 27.7% (for E-Fb1 and E-Fb3, respectively). For the Blask hybrid and the late hybrid Iman, faba bean was less competitive and the sowing rate of the faba bean species had a similar effect on the percentage composition of total yield, from 11.7 to 19.9% (for L-Fb1 and M-Fb3, respectively).

**Figure 8:**
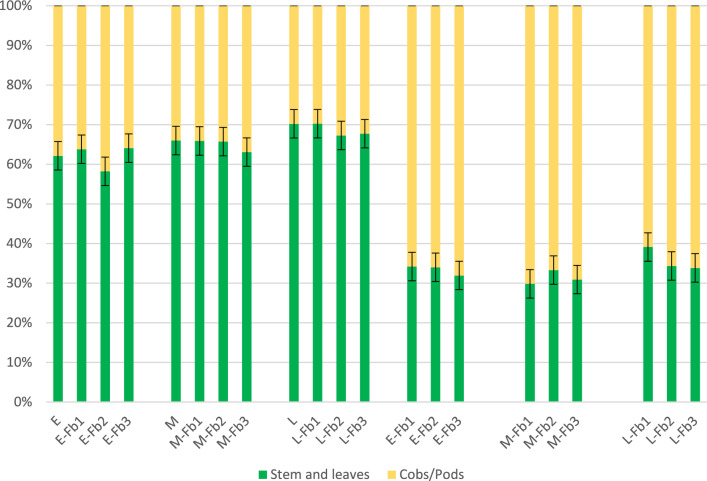
Structure of intercropping biomass components. Percentage of maize and faba bean fresh biomass.

In the early hybrid, the percentage of cobs was highest, ranging from 36 (E-Fb1 and E-Fb3) to 42% (E-Fb2) (Fig. 8). The Blask hybrid featured an even proportion of cobs (34%) except for that obtained from intercropping with faba bean with the highest proportion of faba bean species, which was 37% (M-Fb3). With the Iman hybrid, the proportion of cobs was the lowest: 30–33% (for L-Fb1 and L-Fb2, respectively). For faba bean, the percentage of pods was almost twice as high as the percentage of cobs in maize, ranging from 61 (L-Fb1) to 70% (M-Fb1). There was a noticeable effect of faba bean sowing rate on the percentage of pods in the yield for the early and late hybrids. With the Blask hybrid, no such regularity was found.

### Effect of intercropping system on maize and faba bean protein content

**Figure 9:**
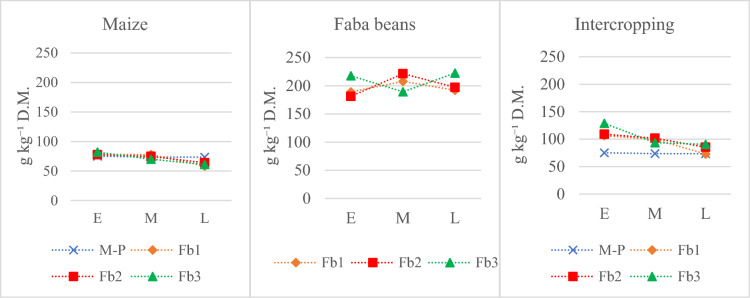
Crude protein concentration (g kg^−1^ D.M.) for maize and faba bean and weighted average. Means from 2005 to 2007. M-P—maize pure sowing; E—early hybrid; M—medium hybrid; L—late hybrid; Fb1—faba beans, sowing rate 18 seeds m^−2^; Fb2—faba beans, sowing rate 27 seeds m^−2^; Fb3—faba beans sowing rate 36 seeds m^−2^.

The characteristics and composition of biomass and the share of cobs and pods in the biomass influenced protein content (Fig. 9). For pure sowing, maize biomass protein content ranged from 61.1 (L-Fb1) to 82.1 g kg^−1^ DM (E-Fb3); while in faba bean biomass, this ranged from 181.3 to 225 g kg^−1^ DM. The weighted average protein content calculated from the proportion of dry matter yield was higher for the intercropping system than in pure maize sowing. Only with the Iman hybrid sown with faba bean (L-Fb1) was the protein content lower than in pure sowing (by 0.6 g). Intercropping the early maize hybrid Wilga with faba bean resulted in a 41–71% higher protein content compared to pure maize biomass. For the Blask hybrid, intercropping increases in protein content ranged from 27 to 38%. The late maize hybrid grown with faba bean at L-Fb2 and L-Fb3 had a 16–23% higher protein content than the pure Iman hybrid biomass.

### Effect of an intercropping system with maize and faba beans on energy and protein yield

**Figure 10:**
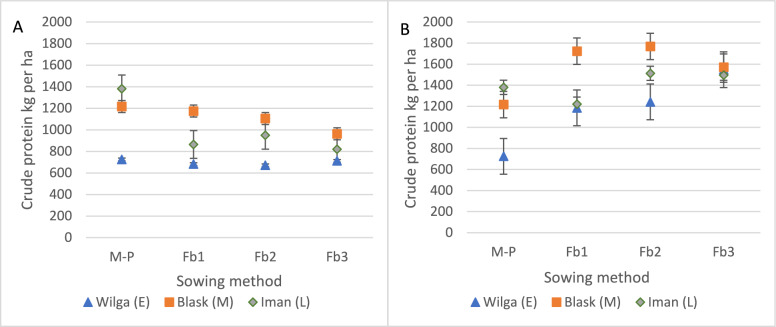
Total protein of maize (**A**) and intercropping maize with faba bean (**B**) (t per ha). Interaction of hybrids and sowing method. Means for 2005–2007.

When cultivating maize hybrids in pure sowing, the highest protein yield was obtained from the Iman hybrid (1380 kg per ha; Table 3, Fig. 10A). In intercropping with faba bean, irrespective of sowing rate, the Blask hybrid provided the highest protein yield: from 963 kg per ha (M-FB3) to 1174 kg per ha (M-Fb1). The interaction of hybrids with faba bean sowing rates was confirmed statistically. Irrespective of the sowing method, the Blask hybrid produced the highest protein yield (1,114 kg per ha), and this was 11% higher than that of the late and 60% higher than that of the early hybrid. Hybrid selection led to no significant differences in protein yield from faba bean.

**Table 3:** Variance analysis of total protein yield and energy yield.

Treatments	Protein yield			Energy yield		
	Maize	Faba bean	Total	Maize	Faba bean	Total
	Sum of squares			Sum of squares		
Year	3,066,893	165,570	3,238,795	3.67E+08	1.25E+07	2.44E+08
Sowing methods	1,505,272	9,134,280	4,171,569	1.69E+08	1.65E+08	6.22E+06
Hybrids	4,464,713	8513	3,772,732	1.21E+09	9.13E+05	1.15E+09
Year × sowing methods	1,782,080	80,158	2,195,064	2.14E+08	5.28E+06	1.99E+08
Year × hybrids	1,694,107	203,523	2,005,821	1.57E+08	3.20E+06	1.31E+08
Sowing methods × hybrids	1,337,657	206,136	2,886,514	5.92E+07	1.95E+06	6.90E+07
Year × sowing methods × hybrids	1,183,019	392,301	2,158,643	1.39E+08	3.92E+06	1.39E+08

Treatment	Mean square			Mean square		
Year	1,533,446	82,785	1,619,397	1.84E+08	6.26E+06	1.22E+08
Sowing methods	501,757	3,044,760	1,390,523	5.62E+07	5.51E+07	2.07E+06
Hybrids	2,232,357	4257	1,886,366	6.05E+08	4.56E+05	5.77E+08
Year × sowing methods	297,013	13,360	365,844	3.57E+07	8.81E+05	3.32E+07
Year × hybrids	423,527	50,881	501,455	3.93E+07	7.99E+05	3.27E+07
Sowing methods × hybrids	222,943	34,356	481,086	9.87E+06	3.25E+05	1.15E+07
Year × sowing methods × hybrids	98,585	32,692	179,887	1.16E+07	3.26E+05	1.15E+07

Treatment	F value			F value		
Year	95.9	6.8	55.6	59.2	28.7	38.0
Sowing methods	31.4	251.6	47.7	18.1	251.9	0.6
Hybrids	139.6	0.4	64.7	195.1	2.1	179.7
Year × sowing methods	18.6	1.1	12.6	11.5	4.0	10.3
Year × hybrids	26.5	4.2	17.2	12.7	3.7	10.2
Sowing methods × hybrids	13.9	2.8	16.5	3.2	1.5	3.6
Year × sowing methods × hybrids	6.2	2.7	6.2	3.7	1.5	3.6

Treatment	*p* value			*p* value		
Year	< 0.001	< 0.01	< 0.001	< 0.001	< 0.001	< 0.001
Sowing methods	< 0.001	< 0.001	< 0.001	< 0.001	< 0.001	0.587
Hybrids	< 0.001	0.704	< 0.001	< 0.001	0.129	< 0.001
Year × Sowing methods	< 0.001	0.365	< 0.001	< 0.001	< 0.01	< 0.001
Year × hybrids	< 0.001	< 0.01	< 0.001	< 0.001	< 0.01	< 0.001
Sowing methods × hybrids	< 0.001	< 0.05	< 0.001	0.382	0.190	0.207
Year × sowing methods × hybrids	< 0.001	< 0.01	< 0.001	< 0.001	< 0.138	< 0.001

**Figure 11:**
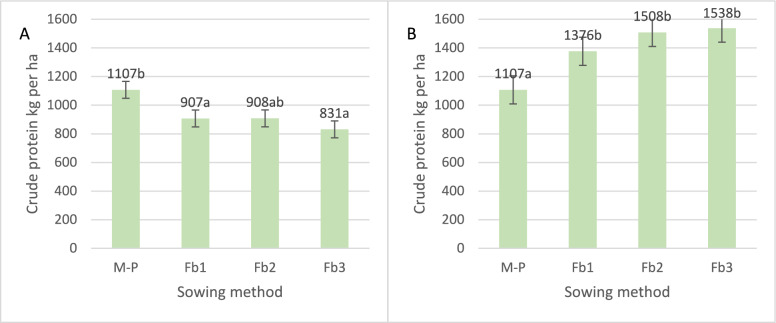
Total protein yield of maize (**A**) and intercropping maize with faba bean (**B**) (t per ha). Average for sowing method. Means for 2005–2007.

On average, a significant decrease in maize hybrid protein yield was found with increasing faba bean crop sowing rates (Fig. 10A). An inverse significant relationship was found for the total protein yield from faba bean (Fig. 10B). Significantly higher protein yield provided in pure maize sowing 1107 kg per ha (Fig. 11A). Overall, the total protein yield from both intercropping components was higher than that from pure maize: from 24% for Fb1 to 39% for Fb3 (Fig. 11B).

**Figure 12:**
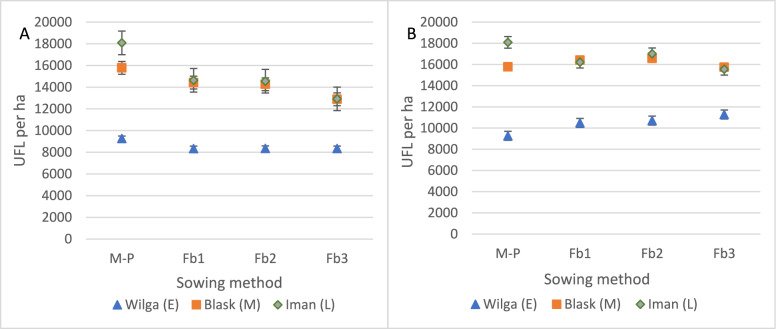
Energy yield in UFL of maize (**A**) and intercropping maize with faba bean (**B**) (UFL per ha). Interaction between hybrids and sowing method. Means for 2005–2007.

**Figure 13:**
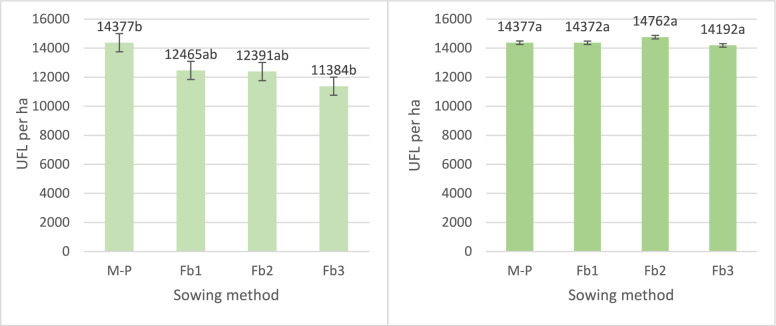
Energy yield in UFL of maize (**A**) and intercropping maize with faba bean **(B**) (UFL per ha). Average for sowing method. Means for 2005–2007.

There was no interaction between hybrids and sowing method with respect to energy yield from maize, faba bean, or total yield from both components (Table 3). On average for each sowing method, increasing the faba bean sowing rate resulted in a significant decrease in UFL maize yield regardless of the hybrid: by as much as 20% (Fb3; Fig. 12A). Faba bean UFL yield compensated total energy yield for early hybrid (regardless of faba beans sowing rate), for medium and late hybrid only Fb1 and Fb2 (Fig. 12B). This was compensated for by an increase in the energy yield from faba bean from 11,384 UFL per ha (Fb3 in maize pure sowing; Fig. 13A) to 14,192 UFL per ha for (Fb3 in intercropping system; Fig. 13B). In pure sowing, Iman yielded 75% more UFL units than Wilga. In intercropping with faba beans, the difference in UFL yield between these hybrids was smaller and amounted to 60%.

**Figure 14:**
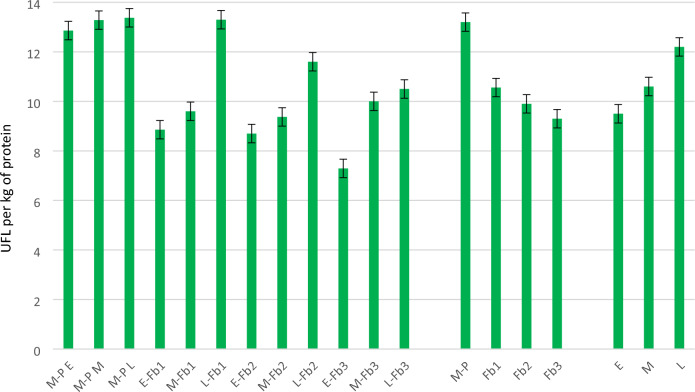
Index of energy–protein ratio. Number of UFL per^1^ kg of protein.

In pure sowing, irrespective of the hybrid, maize was found to accrue approximately 12.5–13.0 UFL per kg of accumulated protein (Fig. 14). In intercropping, the energy–protein balance was affected by the choice of hybrid and the sowing rate of faba bean. Increasing the faba bean sowing rate resulted in an 18% reduction in the UFL-protein balance in the earliest hybrid (from 8.9 to 7.3 units per kg protein). In the medium hybrid, there was a 4% increase, but this rate was still lower than that in pure maize sowing. Intercropping of the late hybrid led to a 20.5% decrease in the energy–protein index.

A maize–faba bean intercropping system influenced biomass structure and chemical composition and is beneficial for the forage protein–energy balance. On average for the sowing methods, a reduction of almost 30% in the energy–protein index can be achieved by introducing faba bean into the maize inter-row compared with pure sowing. With respect to proper forage balancing, the selection of a hybrid for intercropping is also important. The value of the index decreased from 12.2 in the late hybrid to 9.5 in the early hybrid, i.e. a fall of 28%.

## Discussion

Together with intra- and interspecific competition, the selection of species used for intercropping has a crucial significance for intercropping systems^27,28^. In this study, faba beans and maize manifested similar growth rates at the early development stages, up to one-third of the vegetation period. Both species achieved a similar plant growth dynamic and plant height within this period. From the middle of June, maize started intensive growth, while for faba bean the growth dynamic reduced and stabilized at a height of 80–90 cm. At later growing stages, the effect of interspecific competition on maize plant height was found especially in the late hybrid. Thilakarathna et al.^29^ showed that maize in intercropping systems was characterized by stronger intraspecific competition than interspecific. Additionally, access to nitrogen fixed legume plant bacteria via a process of symbiosis fixed by symbiosis bacteria of legumes plants and increased the soil nitrogen pool for maize.

In our research, maize plants grown in a pure sowing system were highest on the last measurement date, irrespective of the hybrid; they were lowest when sown with faba bean at 36 seeds per 1 m^2^. The inhibitory effect of faba bean plants on maize was also demonstrated by Pierre et al.^30^. Similarly, maize in intercropping systems with faba bean species was lower than in pure sowing. In a study by Oskoii et al.^31^, maize plants were 22.5% shorter in intercropping with faba bean than in pure sowing. In our study, faba bean sowing rate showed no effect on maize height. Similarly, no differences in faba bean height were found under the influence of the maize hybrid.

Faba bean was characterized by a shorter vegetation period and at harvest was at full maturity, while maize was at milk-dough maturity stage. Similar results were presented by Liszka-Podkowa and Sowiński^32^, where a faba bean crop reached full maturity at harvest. The dry matter content of faba bean was about 90%, which allows for earlier harvesting when maize biomass has a higher moisture content or is cultivated with a later maize hybrid. In an intercropping system of maize with faba beans, dry mater content increased compared to pure maize biomass: 3.1 to 4.9 percentage points (p.p.) at harvest period for silage; in silage (a few months later), no differences were found in DM^33^.

The maize biomass yield was dependent on the hybrid selection as well as the sowing method. From the early hybrid and from maize intercropped with faba bean sown at 36 seeds per m^2^, biomass yield was on average significantly lower. For faba bean, crop biomass yield—taking into account—the sowing method typically had no significant effect. In an experiment with similar sowing rates for maize (85,000 per ha) and faba bean (350,000 per ha), Stoltz et al.^33^ showed a 37.3% decrease in maize yield and a 2.5% decrease in the total yield of both components compared to pure maize sowing. After the application of N fertilizer (60 kg per ha) in the intercropping, there was an increase in maize yield and an increase in the total yield of 7.2% (but this was non-significant). A similar result—a 4.4–5.2% increase in maize biomass yield and an 8.8–11.3% increase in faba bean biomass yield compared to pure sowing—was reported in a relay cropping system by Li et al.^18^. In our study, the average increase in dry matter yield in the intercropping was lower: ranging from 1.0 to 4.2% compared to maize grown in pure sowing.

In this study, the selection of the hybrid for the intercropping system was found to have a strong effect on biomass production. The early maize hybrid was the most suitable for intercropping and responded to cultivation with faba bean with the highest yield increase (up to 25%). Stoltz et al.^34^ also selected an early maize hybrid for an intercropping system with faba beans for silage. In the late maize hybrid, intercropping with faba beans contributed to an up to 12% reduction in total dry matter yield compared to pure sowing. Intercropping sowing methods should not be recommended for late hybrids for both future research and practice. Under proper soil humidity conditions or farms with irrigation systems, early maize hybrids can be sown as a second crop during the growing season, e.g. after early potatoes, winter barley or some field vegetable crops such as pea for green seeds.

The weighted average protein content of the biomass from the intercropping was 25–57% higher than that of maize in pure sowing. During the intercropping of maize with alfalfa, Liu et al.^35^ obtained an increase in protein content of 31–59%, while in an intercropping of maize with soybean the protein content increased on average by 22% compared to maize in pure sowing^36^. The higher protein content of the intercropped biomass positively influenced protein yield. In the present study, there was a significant interaction between the sowing rate of faba beans and the maize hybrid. In the maize early hybrid, increasing the faba bean sowing rate resulted in a successive increase in protein yield (up to 113% at E-Fb3 compared to pure sowing of an early hybrid) while in the medium and late hybrids, the highest protein yield was obtained at 27 faba bean seeds per m^2^. These results suggest the recommendation of a specific sowing rate for a selected hybrid. A high degree of legume competition in later growing stages mostly decreases maize biomass and protein yield of late maize hybrid. On average, protein yield increased in the intercropping system by 24% to 39% compared to pure maize. Javanmard et al.^37^ showed an increase of 180% in protein yield from intercropping maize with winter vetch compared to that from maize in pure sowing. Stoltz and Nadeau^22^ and Stoltz et al.^33^ achieved a protein yield increase from intercropping systems that was much smaller (7–39%) than that from maize pure sowing.

Balancing ruminant feed energy and protein is a complex issue^38,39^ dependent on physiological changes, the productive potential of the animals, their age and health, and also the quality of the forage itself. In our studies, UFL to protein ratios ranged from 1:77 (maize in pure sowing) to 1:136 g (E-Fb3). In the research conducted by Brun-Lafleur et al.^38^, animals were fed forages with different energy–protein ratios (UFL:PDIE) (i.e. PDIE—protein digested in the small intestine). Ratios ranged from 1:81 to 1:113. Increasing the amount of protein in the feed ration had a positive effect on milk production, protein content, and daily protein and fat yield. In the barn, protein was usually supplemented with concentrates based on soybean meal^38^. The question that arises is whether there would be a similar feed effect with the use of forages obtained from maize–faba beans intercropping.

## Conclusions

Cultivation of faba bean with an early maize hybrid is recommended. This is due to the large differences in the length of the growing season and developmental stage of both components at the time of harvest. Harvesting of biomass from intercropping at the milk-dough maturity stage of maize can be deferred. Intercropping maize with faba bean may provide an alternative, sustainable method to increase protein sources for ruminants under temperate climate conditions. Research should continue to determine the effect on production in animals of increasing the protein content of forage. Sowing in adjacent rows did not have a negative effect on initial maize growth; nonetheless, the results obtained later in the growing season indicate the need to select an appropriate hybrid for intercropping methods. It is also necessary to improve cultivation technology and especially management of weed control. An important issue is to assess the possibility of reducing or eliminating the nitrogen fertilization for intercropping of faba bean cultivation with maize.

## Data Availability

The datasets used and/or analysed during the current study available from the corresponding author on reasonable request.
