# Author Correction: Intercropping maize (*Zea mays* L.) and field beans (*Vicia faba* L.) for forage, increases protein production

**DOI:** 10.1038/s41598-024-75134-5

**Published:** 2024-10-09

**Authors:** Józef Sowiński

**Affiliations:** Institute of Agroecology and Crop Production, Wroclaw University of Environmental and Life Sciences, 50‑375 Wrocław, Poland

Correction: *Scientific Reports* 10.1038/s41598-024-67091-w, published online 16 July 2024

In the original version of this Article, the Funding section was incomplete.

“The research is co-financed from the subsidy increased by the minister responsible for higher education and science for the period 2020–2026 in the amount of 2% of the subsidy referred to Art. 387 (3) of the Act of 20 July 2018—Law on Higher Education and Science, obtained in 2019.”

now reads

“The research is co-financed from the subsidy increased by the minister responsible for higher education and science for the period 2020–2026 in the amount of 2% of the subsidy referred to Art. 387 (3) of the Act of 20 July 2018—Law on Higher Education and Science, obtained in 2019. APC is financed by Wrocław University of Environmental and Life Sciences.”

The original Article has been corrected.

